# Surface PD-L1, E-cadherin, CD24, and VEGFR2 as markers of epithelial cancer stem cells associated with rapid tumorigenesis

**DOI:** 10.1038/s41598-017-08796-z

**Published:** 2017-08-29

**Authors:** Goodwin G. Jinesh, Ganiraju C. Manyam, Chinedu O. Mmeje, Keith A. Baggerly, Ashish M. Kamat

**Affiliations:** 1 Department of Urology, The University of Texas MD Anderson Cancer Center, Houston, Texas 77030 USA; 2 Department of Bioinformatics, The University of Texas MD Anderson Cancer Center, Houston, Texas 77030 USA

## Abstract

Cancer cells require both migratory and tumorigenic property to establish metastatic tumors outside the primary microenvironment. Identifying the characteristic features of migratory cancer stem cells with tumorigenic property is important to predict patient prognosis and combat metastasis. Here we established one epithelial and two mesenchymal cell lines from ascites of a bladder cancer patient (i.e. cells already migrated outside primary tumor). Analyses of these cell lines demonstrated that the epithelial cells with surface expression of PD-L1, E-cadherin, CD24, and VEGFR2 rapidly formed tumors outside the primary tumor microenvironment in nude mice, exhibited signatures of immune evasion, increased stemness, increased calcium signaling, transformation, and novel E-cadherin–RalBP1 interaction. The mesenchymal cells on the other hand, exhibited constitutive TGF-β signaling and were less tumorigenic. Hence, targeting epithelial cancer stem cells with rapid tumorigenesis signatures in future might help to combat metastasis.

## Introduction

Metastasis is the primary cause of cancer-associated mortality^1^. For metastasis to occur, cancer cells must migrate out of the primary tumor microenvironment, efficiently evade the immune system, and establish tumors at distant sites. In most types of cancer, cancer stem cells have been demonstrated to exhibit tumorigenic and immune evasive properties required for metastasis^2^. Bladder cancer occurs in approximately 74,000 patients annually in the US^3^. Approximately 25% of patients present locally advanced or metastatic disease. The standard treatment for patients with locally advanced disease is chemotherapy followed by surgical extirpation, which provides many patients a chance for cure; however, metastasis remains the prime cause of cancer-associated mortality^3^. Recently, immunotherapy with anti-PD-1 therapies have been approved in this setting as well. Hence understanding the molecular and genetic signatures that help cancer cells to evade immune surveillance and establish tumors at distant sites is necessary to predict patient prognosis, develop therapeutics and to combat metastasis.

Migration, metastasis, and stemness of cancer stem cells has been linked to epithelial to mesenchymal transition (EMT)^4^. However, the direct role of EMT in tumorigenesis is not completely understood, and whether metastatic cells undergo mesenchymal to epithelial transition (MET) is not known^5^.

Here we established three cell lines, one epithelial and two mesenchymal, from ascitic fluid of a bladder cancer patient and demonstrated that epithelial cells with surface expression of PD-L1, E-cadherin, CD24, and VEGFR2, transforming phenotype, and E-cadherin-RalBP1 interaction were capable of more rapid tumorigenesis than the mesenchymal cells with constitutively active TGF-β signaling. Our study also reveals genetic signatures and other distinguishing characteristics of migrating cancer stem cells associated with rapid tumorigenesis and lays a foundation for future studies to combat metastasis in bladder cancer.

## Results

### Epithelial cancer cells from ascitic fluid form tumors more rapidly than mesenchymal cancer cells from ascitic fluid

**Figure 1:**
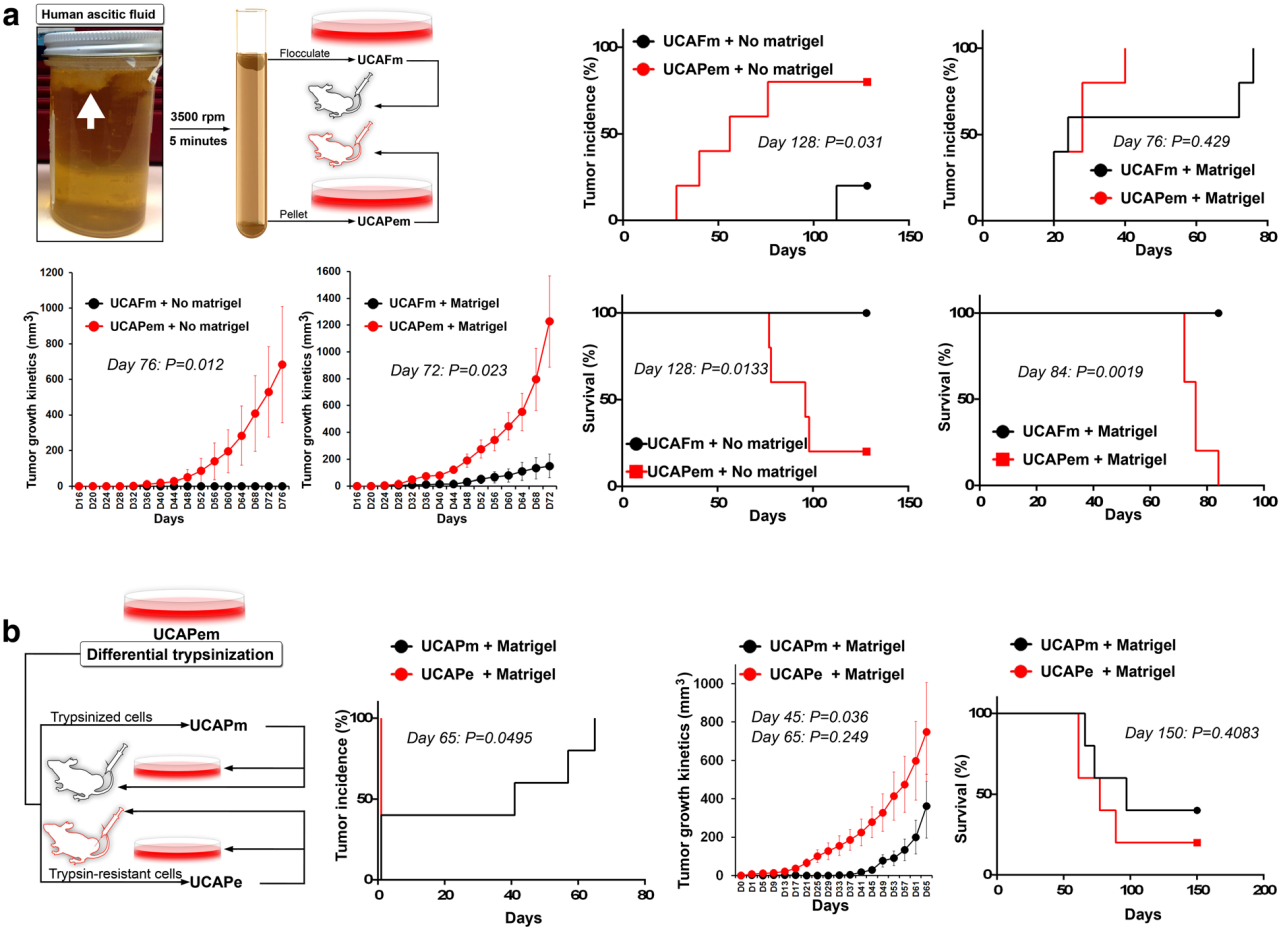
Epithelial cancer cells from ascitic fluid form tumors more rapidly than mesenchymal cancer cells from ascitic fluid. (**a**) Ascitic fluid from a bladder cancer patient had massive amount of flocculated cells (top left panel, arrow) that were separated from pelletable cells by centrifugation. Flocculated cells, which had mesenchymal properties on microscopic examination (UCAFm cells), and pelleted cells, which had both epithelial and mesenchymal properties on microscopic examination (UCAPem cells), were evaluated with or without matrigel for tumorigenicity (top right panels), tumor growth kinetics (bottom left panels), and survival (bottom right panels) in nude mice (n = 5). (**b**) UCAPem cells were segregated into cells with epithelial morphology (UCAPe) and cells with mesenchymal properties (UCAPm) by differential trypsinization (first panel), and these subtypes were evaluated with matrigel for tumorigenicity (second panel), tumor growth kinetics (third panel), and survival (fourth panel) in nude mice. Tumor growths had significant difference on day 45 but not on day 65 (n = 5).

Migrating cancer cells require tumorigenic potential to establish metastasis. To characterize the tumorigenicity of cancer cells that had migrated out of the primary tumor microenvironment, we collected ascitic fluid from a bladder cancer patient (under IRB approval, please see Materials and Methods for clinical details). The ascitic fluid collected contained a major proportion of flocculated cells, which were separated from pelletable cells by centrifugation. Microscopic examination revealed that the flocculated cells had mesenchymal morphology and the pelleted cells were a mixture of cells with epithelial and mesenchymal morphology. On the basis of these findings, we named the flocculated cells as urothelial carcinoma ascitic-fluid flocculate cells with mesenchymal morphology (UCAFm cells) and the pelleted cells as urothelial carcinoma ascitic-fluid pellet cells with mixture of epithelial and mesenchymal morphology (UCAPem cells) (Fig. 1a). Tumorigenicity assays in nude mice revealed that UCAPem cells gave rise to more tumors than UCAFm cells and that the tumors from UCAPem cells grew more rapidly and were associated with a worse prognosis than tumors from UCAFm cells (Fig. 1a). We further separated the UCAPem cells by differential trypsinization to obtain cells with mesenchymal morphology (UCAPm; relatively trypsin sensitive) and cells with epithelial morphology (UCAPe; relatively trypsin resistant). Tumorigenicity assays in nude mice revealed that tumors from UCAPe cells developed more rapidly than tumors from UCAPm cells but that the two tumor types exhibited no significant differences in tumor growth kinetics or prognosis (Fig. 1b).

### Validation of epithelial and mesenchymal phenotypes of UCAFm, UCAPm, and UCAPe cells with established EMT genetic signatures

**Figure 2:**
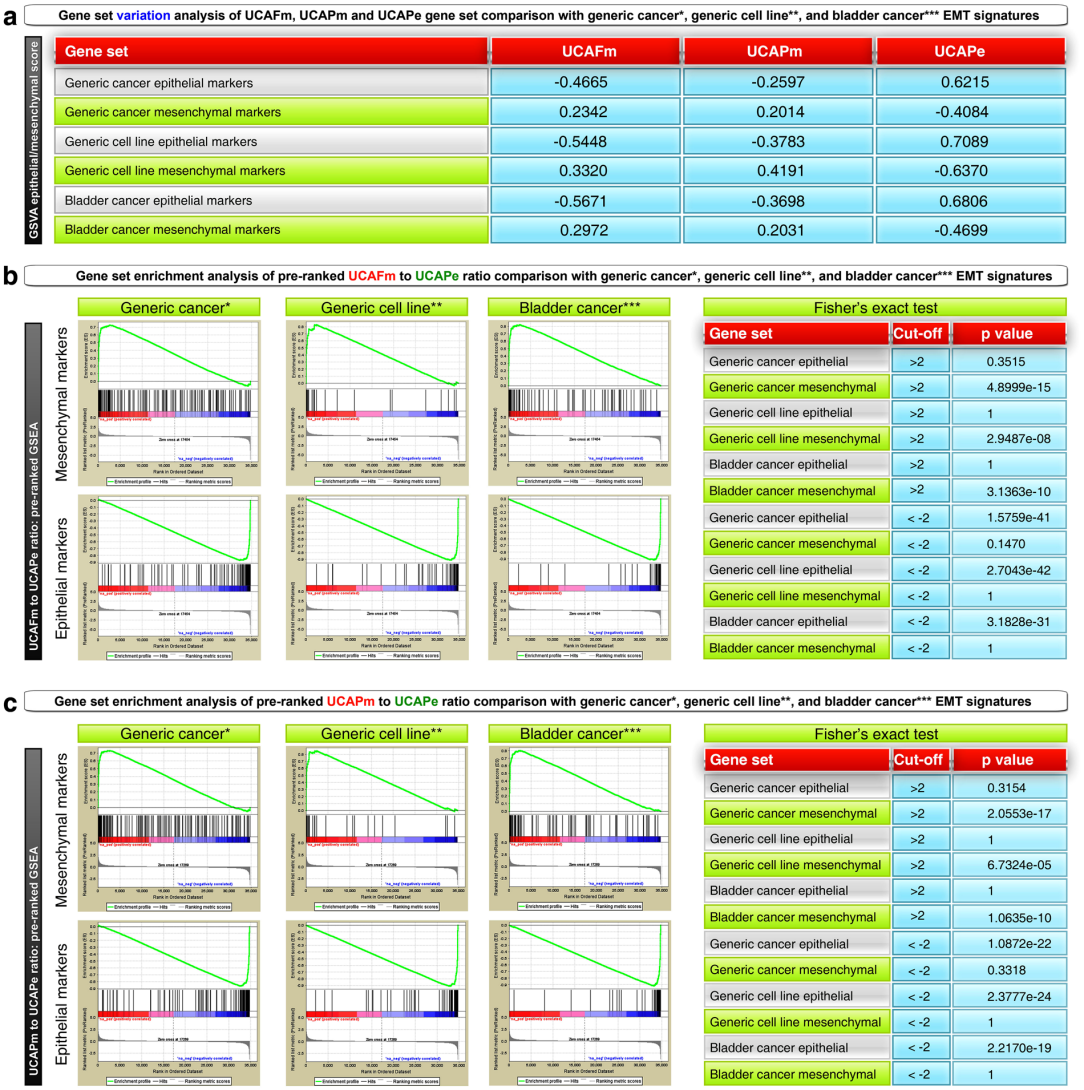
Gene expression profiling reveals that only UCAPe cells are epithelial. (**a–c**) Gene expression profiles of UCAFm, UCAPm, and UCAPe cells were compared with generic cancer (*based on 6 cancer types: see text for reference), generic cell line (**based on cell lines from multiple cancer types: see text for reference), and bladder cancer (*** based on bladder cancer specimens: see text for reference) EMT signatures. (**a**) Gene set variation analysis (GSVA) of UCAFm, UCAPm, and UCAPe cells individually shows that only UCAPe cells had positive epithelial scores whereas UCAFm and UCAPm cells had positive mesenchymal scores. (**b**) Gene set enrichment analysis (GSEA) of pre-ranked UCAFm to UCAPe gene ratio, compared with established generic cancer, generic cell line, and bladder cancer EMT signatures. Enrichment map is shown on left. Fisher’s exact test p values are shown on right to validate significance. (**c**) Gene set enrichment analysis (GSEA) of pre-ranked UCAPm to UCAPe gene ratio, compared with established bladder cancer, generic cell line, and generic cancer EMT signatures. Enrichment map is shown on left. Fisher’s exact test p values are shown on right to validate significance.

Since UCAPe cells with epithelial morphology formed tumors rapidly compared to mesenchymal UCAFm and UCAPm cells we systematically examined EMT in these cells. We performed whole transcriptome gene expression profiling of these cells and compared the results with established EMT signatures (based on bladder, breast, colorectal, gastric, ovarian, and lung cancers)^6^. Gene set variation analysis (GSVA) with established EMT signatures (generic cancer, generic cell line, and bladder cancer) yielded positive epithelial scores for UCAPe cells and positive mesenchymal scores for UCAFm and UCAPm cells (Fig. 2a). We next compared the genes that showed differences in expression between these cell lines by gene set enrichment analysis (GSEA) and by computing ratios of UCAFm to UCAPe or UCAPm to UCAPe (fold change >2) to identify whether the cell lines were enriched with established EMT signatures (generic cancer, generic cell line, and bladder cancer) by Fisher’s exact test^6^. GSEA and Fisher’s exact test results revealed that the gene expression signatures of UCAPe cells significantly aligned with epithelial signatures of generic cancer, generic cell line, and bladder cancer, whereas the gene expression signatures of UCAFm and UCAPm cells significantly aligned with mesenchymal signatures (Fig. 2b,c). These data confirmed the epithelial nature of UCAPe cells and the mesenchymal nature of UCAFm and UCAPm cells. We also generated the identities of these cell lines by short tandem repeat (STR) DNA fingerprinting (STR profiles were similar because of the cell lines’ origin from the same patient) and found that proliferation rates were similar after an initial lag period of 48 hours (Supplemental Figure 1).

### UCAPe cells differ from UCAFm and UCAPm cells in terms of EMT, stemness, calcium signaling, and immune signatures

**Figure 3:**
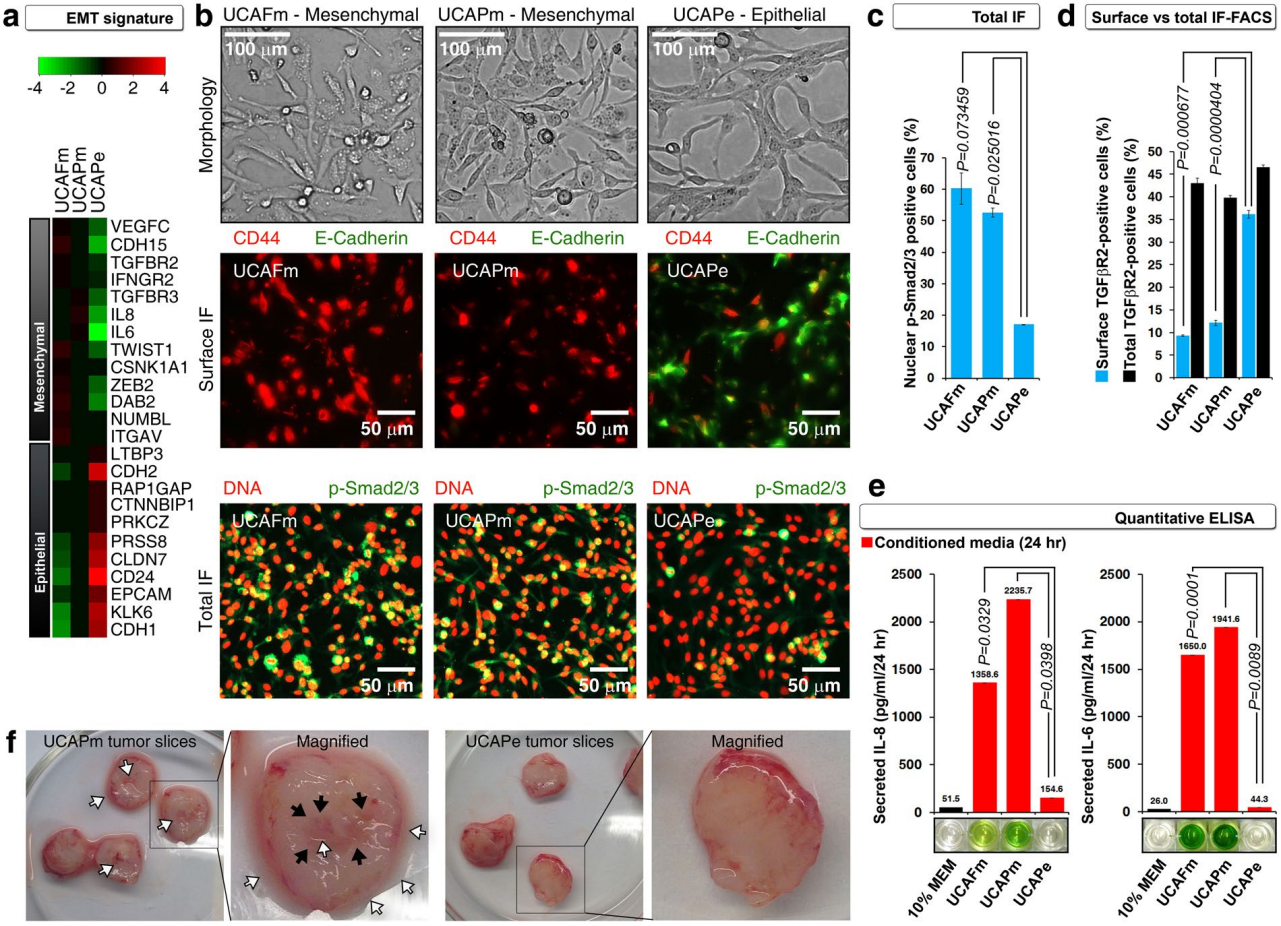
UCAPe cells differ from UCAFm and UCAPm cells in epithelial and mesenchymal phenotype. (**a**) cDNA microarray analysis of selected EMT regulators showing epithelial signature of UCAPe cells and mesenchymal signature of UCAFm and UCAPm cells. See Supplemental Table 1 for references on selected genes and their links to EMT. (**b**) Differences in morphology (top row), E-cadherin expression (middle row), and TGF-β signaling (bottom row) between UCAFm, UCAPm, and UCAPe cells (n = 3). Only UCAPe cells expressed E-cadherin on the cell surface as analyzed by surface immunofluorescence (IF) (n = 3) (middle row). Only UCAFm and UCAPm cells had constitutive TGF-β signaling (assessed by nuclear phospho-Smad2/3; DNA stained by propidium iodide) (n = 3) (lower row). CD44 is a proposed bladder stem cell marker (in normal as well as cancer tissues). (**c**) Quantification of the percentage of cells with nuclear phospho-Smad2/3 staining in panel b (n = 3). (**d**) Quantification of the percentage of cells with basal surface TGF-βR2 by surface IF-FACS and total TGF-βR2 by total IF-FACS (n = 3). (**e**) Quantitative ELISA showing secreted IL-8 and IL-6 (TGF-β target genes) in UCAFm and UCAPm cells but not in UCAPe cells (n = 2). (**f**) Photographs of slices from tumors formed by UCAPm and UCAPe cells in nude mice showing differences in fluidity. Black arrows show loose packing areas and white arrows show fluid in and around the slices.

We further compared expression of the known epithelial and mesenchymal regulator genes in UCAFm, UCAPm, and UCAPe cells (Fig. 3a and Supplemental Table 1). Microarray analysis showed that UCAPe cells had high expression of known epithelial markers, including E-cadherin (encoded by *CDH1*), and poor expression of *ZEB2*, a negative regulator of E-cadherin (Fig. 3a). Furthermore, UCAPe cells had poor expression of the EMT-inducing TGF-β signaling components, including TGF-βR2, and TGF-βR3, and high expression of *LTBP3*, a negative regulator of TGF-β signaling (Fig. 3a). We validated these data by surface immunofluorescence microscopy, which showed surface E-cadherin expression only in UCAPe cells and showed constitutively active TGF-β signaling (indicated by nuclear phospho-Smad2/3) only in UCAFm and UCAPm cells (Fig. 3b,c and Supplemental Figure 1). UCAPm and UCAFm cells but not UCAPe cells exhibited increased internalization of TGF-βR2 and secretion of TGF-β signaling targets related to EMT (IL-8 and IL-6), indicating functional constitutive activation of TGF-β signaling in mesenchymal cells (Fig. 3d,e). Taken together, these data demonstrated that UCAPe cells can be distinguished from UCAPm and UCAFm cells on the basis of UCAPe cells’ high surface E-cadherin expression and poorly active TGF-β signaling. Interestingly, UCAPm cells formed more loosely packed tumors than UCAPe cells, indicating that the cells retained their mesenchymal (UCAPm) or epithelial (UCAPe) characteristics *in vivo* (Fig. 3f).

**Figure 4:**
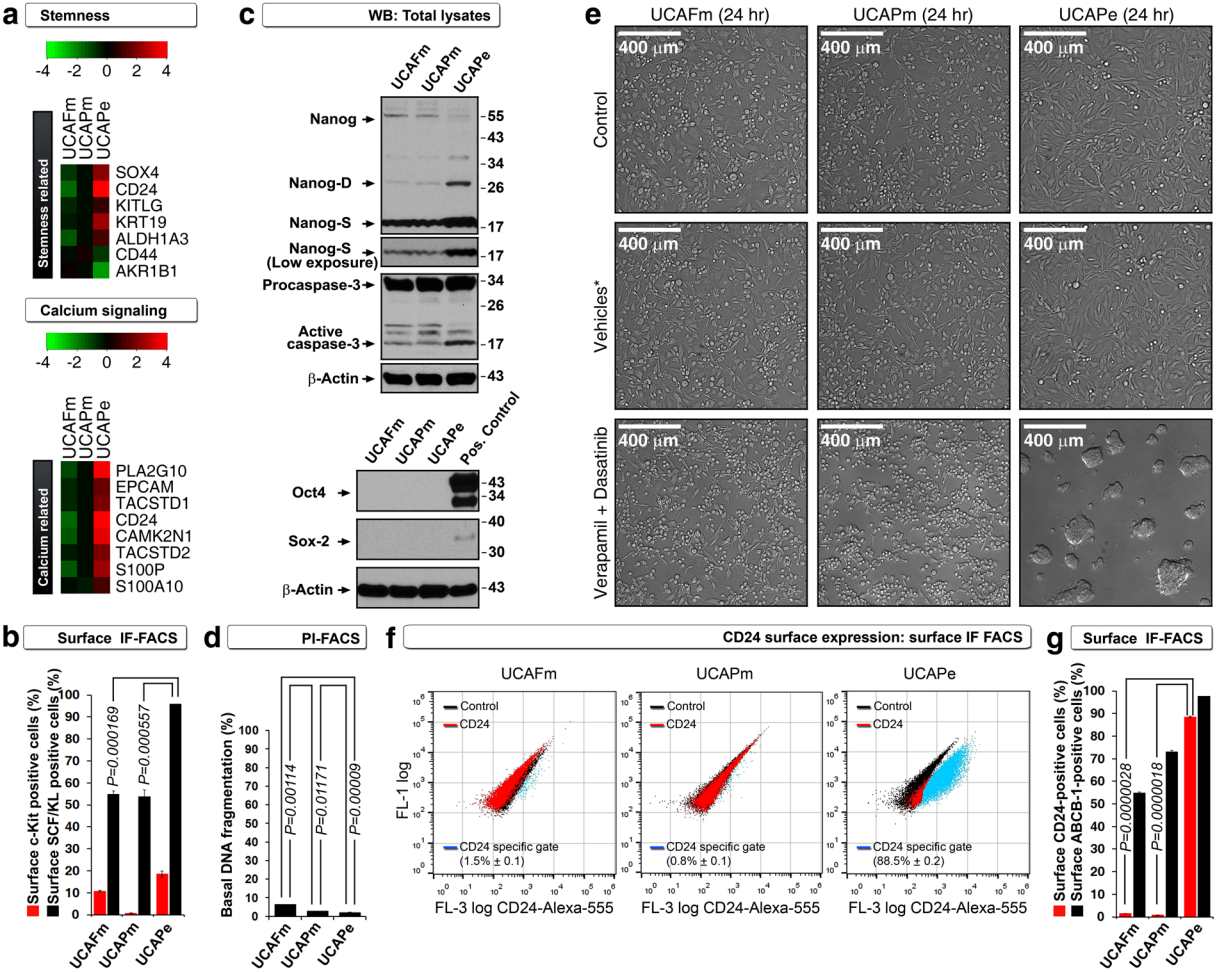
Calcium signaling links stemness and tumorigenicity to the transforming phenotype of epithelial UCAPe cells. (**a**) cDNA microarray analysis showing that stemness and calcium signaling regulators were upregulated in UCAPe cells. See Supplemental Tables 2 and 3 for references on selected genes and their links to stemness and calcium signaling. (**b**) Surface immunofluorescence (IF) FACS showing that surface c-Kit ligand (SCF/KL) but not surface c-Kit discriminated UCAPe cells from mesenchymal cells (n = 3). (**c**) Western blotting (WB) showing that constitutive caspase-3 activation and generation of Nanog-D (cleaved fragment related to cellular differentiation) and Nanog-S (cleaved fragment related to sphere formation) discriminated UCAPe cells from mesenchymal cells (top panel), whereas Oct4 and Sox-2 were not expressed in any of the three cell lines (bottom panel). (**d**) Constitutive caspase-3 activation did not lead to DNA fragmentation evaluated by PI-FACS at 24 hours (n = 3). (**e**) Only UCAPe cells were able to transform in response to calcium signaling inhibition by verapamil plus dasatinib (n = 3). (**f,g**) CD24 but not ABCB1 cell surface expression as evaluated by surface IF-FACS marked transforming UCAPe cells (n = 3).

We next examined stemness of these cell lines because stemness is an important characteristic of cancer stem cells necessary for tumorigenesis^7^. Expression of mRNAs of stem cell markers, including *KRT19*, *SOX4*, *KITLG* (c-Kit ligand), and *ALDH1A3*, were upregulated in UCAPe cells, and c-Kit ligand protein cell surface expression was higher in UCAPe cells than in UCAFm or UCAPm cells (Fig. 4a,b and Supplemental Table 2). To establish the relationship between epithelial phenotype and stemness, we focused on Nanog because E-cadherin-mediated cell-cell contact is required for reprogramming of induced pluripotent cells by Nanog^8^ and immune evasion^9^. Nanog is cleaved by caspase-3 to a 27-kDa fragment during differentiation (Nanog-D)^10^, and cleaved to a 17-kDa fragment during blebbishield-mediated sphere formation (Nanog-S)^11^. Compared to UCAPm and UCAFm cells, UCAPe cells had increased caspase-3 activation and higher expression of Nanog-D and Nanog-S cleavage fragments, but none of these cells expressed other stemness factors, such as Sox-2 and Oct4, or underwent DNA fragmentation due to caspase-3 activation (Fig. 4c,d). These data demonstrated that Nanog contributes to stemness in UCAPe cells more than in UCAFm and UCAPm cells.

We next examined calcium signaling, as E-cadherin-mediated cell-cell contact-induced sphere (bullet) formation/transformation is dependent on calcium^12^. Expression of mRNAs of genes involved in calcium signaling, including *S100A10*, *TACSTD2*, *EPCAM/TACSTD1*, *S100P*, *CAMK2N1*, *CD24*, and *PLA2G10*, were upregulated in UCAPe cells, indicating that this cell line has altered calcium signaling (Fig. 4a and Supplemental Table 3). Of these genes *CAMK2N1* is an inhibitor of calcium signaling; thus, we tested the effect of calcium inhibition using two calcium signaling blockers, verapamil and dasatinib, that target Src and ABCB1/Mdr-1/P-glycoprotein^13^. Verapamil plus dasatinib induced cellular transformation only in UCAPe cells, demonstrating that calcium signaling is linked to transformation in UCAPe cells (each agent on its own also could induce cellular transformation in UCAPe cells; personal observation) (Fig. 4e). In addition, more UCAPe cells were positive for surface ABCB1 and surface CD24 than UCAPm and UCAFm cells (Fig. 4f,g). Thus, calcium signaling inhibition is coupled to the transforming phenotype of UCAPe cells.

**Figure 5:**
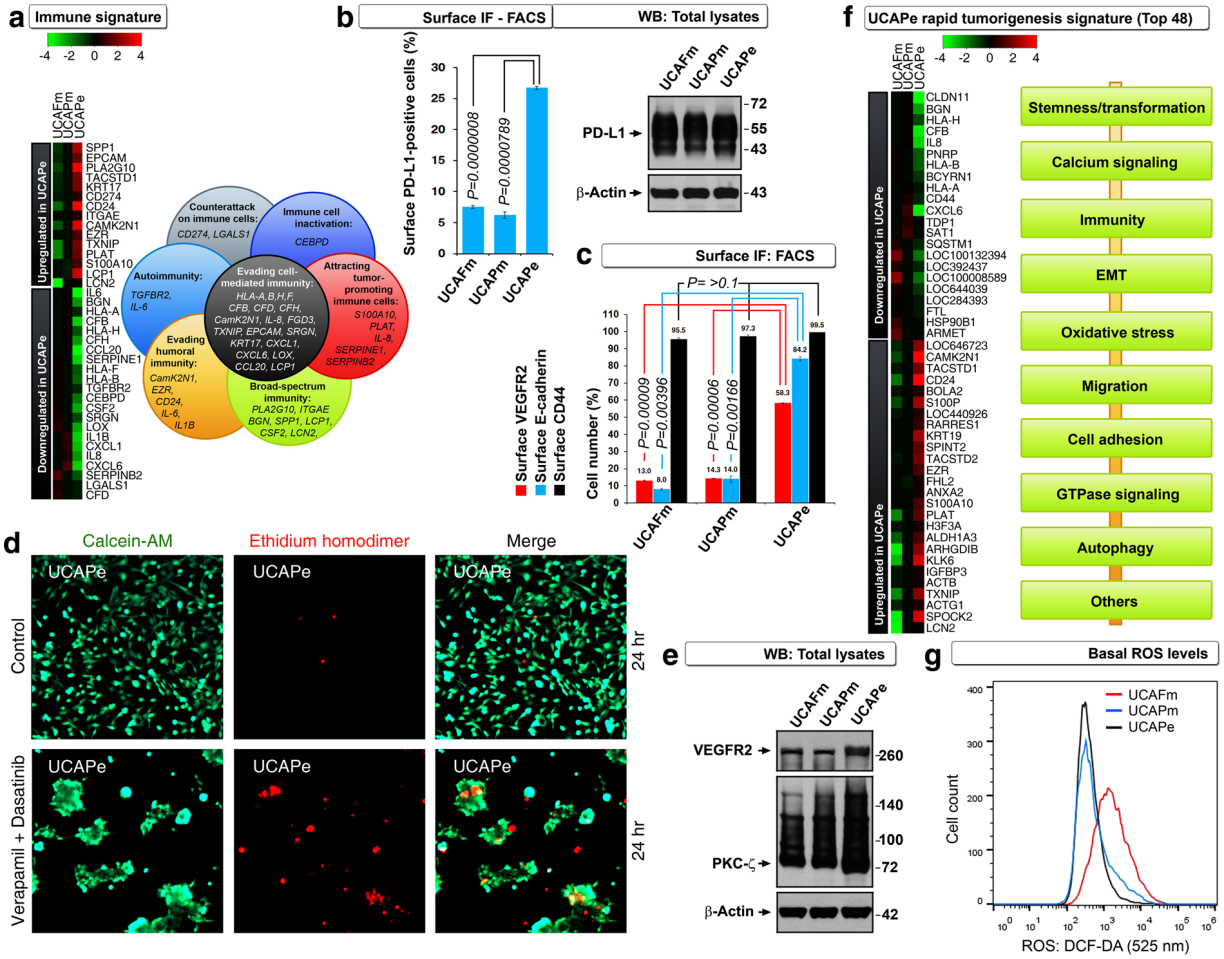
Rapid tumorigenic signatures of UCAPe cells. (**a**) cDNA microarray analysis showing that humoral and cell-mediated immunity–related genes were downregulated and tumor-promoting immune regulators were upregulated in UCAPe cells. See Supplemental Table 4 for references on selected genes and their links to immunity. (**b**) Surface PD-L1 (immunofluorescence [IF] FACS) (n = 3) but not total PD-L1 (Western blot [WB]) discriminated UCAPe cells from mesenchymal cells. (**c**) Surface E-cadherin and VEGFR2 but not CD44 (IF-FACS) discriminated UCAPe cells from mesenchymal cells (n = 3). (**d**) Live (green) or dead/dying (red) assay showing the presence of cell death process within transforming spheres generated by calcium signaling inhibition (n = 3). (**e**) Western blot showing increased VEGFR2 and PKC-ζ expression in UCAPe cells. (**f**) cDNA microarray gene expression analysis showing top 48 genes discriminating UCAPe cells from mesenchymal cells. See Supplemental Table 5 for references on selected genes and their links to groups indicated in boxes at right. (**g**) DCF-DA FACS showing that, higher basal ROS levels marked less tumorigenic UCAFm cells (n = 3).

We next examined the immune signatures of UCAFm, UCAPm and UCAPe cells because bladder cancer responds to immunotherapy and efficient immune evasion is necessary for rapid tumorigenesis and poor prognosis^14–16^. Genes that are linked to cancer counterattack against immune cells, inactivation of immune cells, attraction of tumor-promoting immune cells, evasion of cell-mediated immunity, evasion of humoral immunity, and autoimmunity were selectively altered in UCAPe cells (Fig. 5a and Supplemental Table 4). Of these genes, we validated *CD274*, which encodes PD-L1, an immune tolerance-regulatory molecule from tumor cells. More UCAPe cells expressed PD-L1 on the cell surface than UCAPm or UCAFm cells did, even though these three cell lines expressed equal quantities of total PD-L1 (Fig. 5b). This result is clinically relevant because the findings from a clinical trial in bladder cancer showed differential activity to checkpoint inhibitor based on PD-L1 status^17^.

### Surface VEGFR2 and E-cadherin links cellular transformation in response to calcium channel inhibition

E-cadherin^18^ and VEGFR2^19^ are known to drive transformation and E-cadherin-mediated calcium signaling is known to enhance VEGF signaling^12^. We found that surface expression of VEGFR2 and E-cadherin but not CD44 discriminates UCAPe cells from UCAPm and UCAFm cells (Fig. 5c). Cancer stem cells are known to undergo transformation by blebbishield formation after commitment to apoptosis^11, 19–25^, and VEGFR2 is required for sphere formation/transformation after apoptosis^19, 21, 26^. Hence, we examined the process of cell death during calcium channel blockade–induced transformation. We found that UCAPe cells indeed exhibited cell death process during transformation (Fig. 5d). VEGFR2 transduces calcium signaling through PKC-ζ^27^, and we observed that UCAPe cells expressed more PKC-ζ than UCAPm and UCAFm cells (Fig. 5e). Together, these data linked E-cadherin and VEGFR2 in calcium channel blockade-induced cellular transformation.

### Surface CD24 expression marks epithelial cancer stem cells with a VEGF-VEGFR2-E-cadherin-calcium-RalA-RalBP1-driven tumorigenic engine

In order to identify the key genetic signature of UCAPe cells, we chose the top 48 genes from microarray data that discriminated UCAPe cells from UCAPm and UCAFm cells (on the basis of either upregulation or downregulation) and classified them into groups based on their function (Fig. 5f and Supplemental Table 5). Importantly, *IGFBP3*, one of the genes upregulated in UCAPe cells, has been implicated in the suppression of oxidative stress to promote tumor growth^28^. Furthermore, highly tumorigenic cells are known to exhibit lower levels of reactive oxygen species (ROS) than less tumorigenic cells^29^. Hence, we examined the baseline ROS levels of these three cell lines. We found that UCAPe and UCAPm cells had lower levels than UCAFm cells (Fig. 5g), and this finding correlated with the prognosis of nude mice with tumors formed from these cell types (Fig. 1).

**Figure 6:**
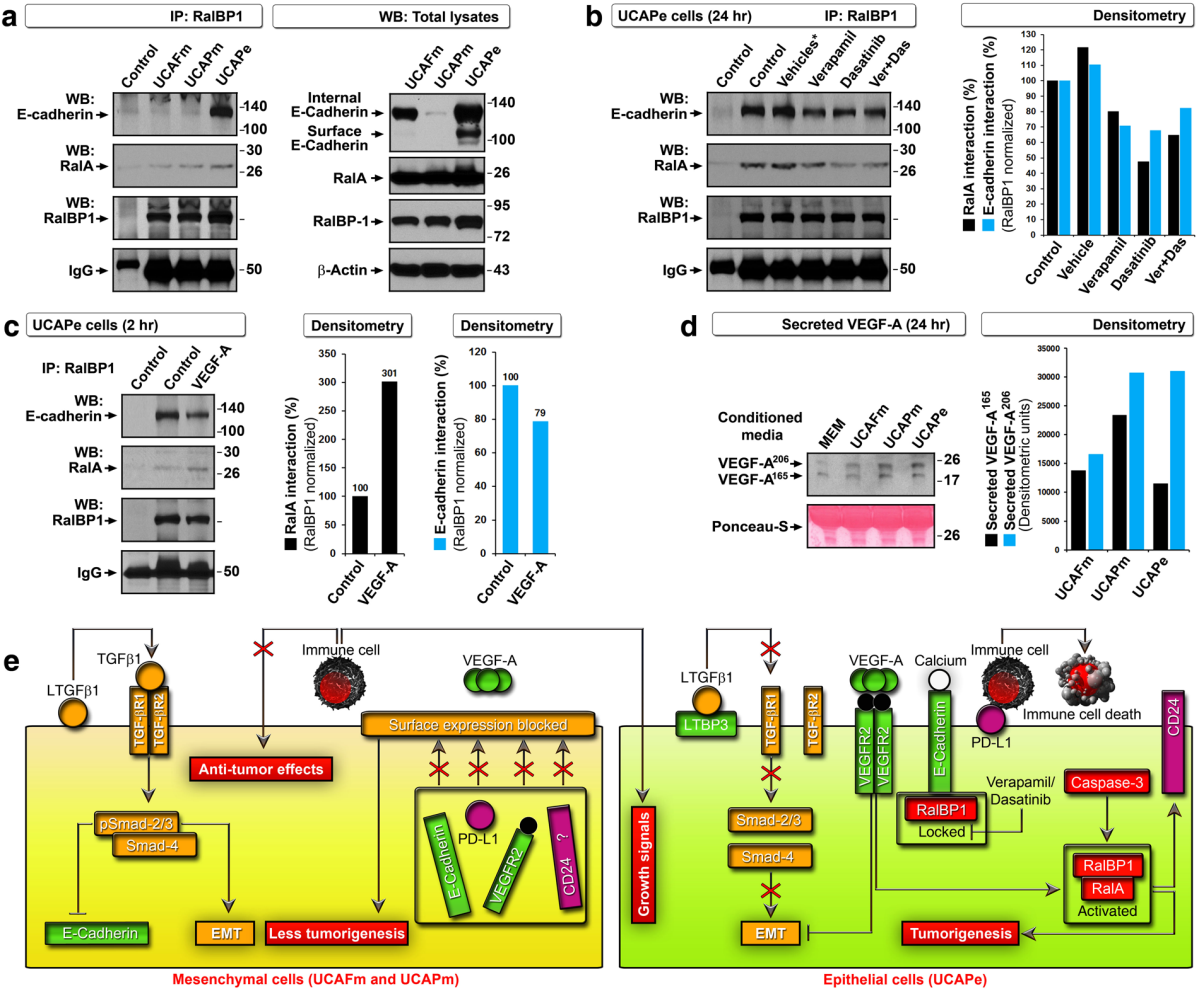
A VEGF-regulated E-cadherin–RalBP1 interaction discriminates epithelial cancer stem cells with rapid tumorigenesis from mesenchymal cells. (**a**) E-cadherin co-immunoprecipitated with RalBP1 only in UCAPe cells with increased RalA binding (left). Total lysates indicate that UCAFm cells expressed both RalBP1 and E-cadherin but the proteins did not interact (right). (**b**) E-cadherin–RalBP1 interaction was reduced in response to calcium signaling inhibition, resulting in reduced RalA activation as evaluated by immunoprecipitation (IP) (left). Densitometric quantification of E-cadherin/RalA to RalBP1 interaction (normalized based on RalBP1) (right). (**c**) IP (left) and densitometry (bar graphs) show that VEGF induced reduced E-cadherin–RalBP1 interaction and increased RalA activation. (**d**) VEGF was secreted from all three cell lines, as evaluated by Western blotting of conditioned media (left). MEM, control medium. Densitometric quantification of secreted VEGF-A isoforms by subtracting control MEM values (right). (**e**) Schematic showing the signaling interplay between EMT regulators and epithelial cancer stem cell markers. Signaling events within mesenchymal cells (left) and epithelial cells (right) are depicted. EMT-related events/molecules are marked in yellow; events/molecules drive epithelial characteristics are marked in green; molecules related to immunity are marked in purple; and events/molecules linked to tumorigenesis/tumor growth are marked in red. LTGFβ1, latent transforming growth factor β1; LTBP3, latent TGF-β binding protein 3. VEGF-A, vascular endothelial growth factor-A; EMT, epithelial to mesenchymal transition; PD-L1, programmed death-ligand-1; TGF-βR1/2, receptors for TGF-β1; VEGFR2, receptor for VEGF-A.

Importantly, *CD24* was consistently ranked in EMT, stemness, calcium signaling, and immunity and was among the top 48 UCAPe-discriminating genes, suggesting an important role for *CD24*. CD24 expression is known to be regulated by RalA-RalBP1 signaling, and RalA-RalBP1 is required for tumorigenesis by human cells^30, 31^. Since RalBP1 is an important endocytosis regulator, we evaluated its association with E-cadherin. Interestingly, even though UCAFm cells and UCAPe cells expressed both RalBP1 and E-cadherin in total lysates, E-cadherin co-immunoprecipitated with RalBP1 only in UCAPe cells (IMEx accession: IM‐24526) (Fig. 6a). Furthermore, RalBP1 expression was higher and RalA activation (interaction with RalBP1) was greater in UCAPe cells than in UCAFm and UCAPm cells (Fig. 6a). We also found that verapamil and dasatinib reduced the E-cadherin–RalBP1 interaction, resulting in reduced RalA activation (Fig. 6b), which indicated that E-cadherin–RalBP1 interaction was calcium dependent.

We next examined how VEGF signaling is related to the interaction of E-cadherin and RalBP1. Recombinant human VEGF reduced E-cadherin–RalBP1 interaction but resulted in increased RalA activation at 2 hours in UCAPe cells (Fig. 6c). It is known in bladder cancer cells that the loss of E-cadherin interaction with RalGDS (an activator of RalA) results in robust endocytosis during blebbishield formation^19^. Thus, our findings of VEGF-induced reduction in E-cadherin–RalBP1 interaction and increase in RalA activation suggested that loss of E-cadherin–RalBP1 interaction is the mechanism by which dasatinib and verapamil induced transformation in UCAPe cells. Notably, all three cell lines were capable of secreting VEGF-A isoforms (Fig. 6d). Together, these data demonstrated that E-cadherin–RalBP1 interaction discriminates UCAPe cells from UCAPm and UCAFm cells in terms of rapid tumorigenesis and CD24, VEGFR2, and E-cadherin surface expression and explain how VEGF-A regulates this interaction to activate the tumorigenic engine.

## Discussion

In this study, we established three cancer cell lines (epithelial UCAPe and mesenchymal UCAFm and UCAPm), from the ascitic fluid of a bladder cancer patient. UCAPe cells differed from the mesenchymal cells by the surface expression of PD-L1, E-cadherin, CD24, and VEGFR2 and exhibited the hallmarks of cancer stem cells, immune evasion, transformation, and in particular, rapid tumorigenesis. We for the first time demonstrate that VEGF, surface VEGFR2 and E-cadherin are linked to RalA/RalBP1 activation with a calcium signaling switch to regulate rapid tumorigenesis of epithelial cells (UCAPe). CD24 is the end product of this signaling event, which is displayed on the surface these cells because RalA/RalBP1 signaling is known to regulate CD24 expression^30^. Our findings are in line with the fact that, the epithelial cells isolated from ascitic fluid of ovarian cancer patients with transforming property act as professional cancer stem cells^32^. Furthermore, E-cadherin^18^ and VEGFR2^19^ are known to drive transformation, and are expressed only on the surface of UCAPe cells to explain why only this cell line could undergo transformation (Figs 4e and 5c).

Both EMT and MET have been implicated in metastasis^33^. However, because we established cells from ascitic fluid (cells that had already migrated out of primary tumor), examining the involvement of MET in tumorigenesis gains more importance than EMT. In contrary to MET, UCAFm and UCAPm cells remained as mesenchymal cells and UCAPe cells remained as epithelial cells (*in vitro*) throughout the course of our studies (∼2 years). These cells might also retain their phenotypes *in vivo* because the UCAPe tumors were more tightly packed than UCAPm tumors (Fig. 3f). Interestingly, VEGF/VEGFR2 is linked to migration^34^, and epithelial cells are capable of migration by forming lamellipodia^35^ without undergoing EMT because VEGFR2 can inhibit EMT^36^ (Fig. 6e). Thus, surface expression of E-cadherin, VEGFR2, PD-L1, and CD24 and transforming phenotype might have played more direct roles in rapid tumorigenesis of UCAPe cells than MET. The interaction of E-cadherin with RalBP1, observed only in epithelial UCAPe cells, and the regulation of RalA-RalBP1 by VEGF and calcium signaling supports this concept (Fig. 6a,c,e). Furthermore, lack of RalBP1–E-cadherin interaction in UCAFm cells (despite their expression of both RalBP1 and internal E-cadherin), with weak tumorigenicity, demonstrates the importance of this interaction in tumorigenesis.

Interestingly, constitutively active TGF-β signaling marked the mesenchymal and less aggressive and/or less tumorigenic cells. This helps to explain why E-cadherin expression is impaired in mesenchymal cells: TGF-β signaling is known to impair E-cadherin expression^32, 37^ (Fig. 6e). Alternatively, TGF-β signaling may have acted as a tumor suppressor pathway in mesenchymal cells^38^. In support of this fact reduced Smad2/3 signaling is known to enhance tumorigenesis^39^. However, the role of TGF-β signaling in immunosuppression cannot be ignored as it is known to block immune attack against cancer^40, 41^. In line with this notion, UCAPem cells, which included both UCAPe and UCAPm cells (with active TGF-β signaling from mesenchymal cells), were able to form aggressive tumors (Fig. 1a).

In conclusion, migrating epithelial cancer stem cells with surface expression of E-cadherin, CD24, PD-L1, and VEGFR2 and transforming phenotype with E-cadherin-RalBP1 interaction are capable of establishing tumors at distant sites. Detection of cancer cells in patients with a rapid tumorigenic signature similar to UCAPe cells might help to predict prognosis, understand metastasis, aid in diagnosis of circulating cancer stem cells, and help to combat metastasis in the future.

## Materials and Methods

### Reagents

Matrigel matrix (356234) was purchased from Corning. Quantikine ELISA kits for IL-6 (D6050) and IL-8 (D8000C), recombinant VEGF-A^165^ (293-VE-010; used at 19 ng/ml). and PE-conjugated VEGFR2 antibody for surface staining (FAB357P; IF-FACS 1:120) were purchased from R&D Systems. Antibodies to CD44 (5640; IF and IF-FACS 1:100), E-cadherin (3195; IF-FACS 1:100; WB 1:1000), Nanog (3580; WB 1:1000), Oct4 (4286; WB 1:1000), Sox-2 (2748; WB 1:1000), SCF/Kit ligand (2093; IF-FACS 1:100), PD-L1 (13684; IF-FACS 1:100), RalA (4799; WB 1:1000), and RalBP1 (5739; WB 1:1000) were purchased from Cell Signaling Technology. Antibodies to phospho-Smad2/3 (Sc-11769R; IF 1:100), TGF-βR2 (Sc-220; IF-FACS 1:100), caspase-3 (Sc-7148; WB 1:500), ABCB1/Mdr1/P-glycoprotein (Sc-8313; IF-FACS 1:100), CD24 (Sc-19585; IF-FACS 1:100), VEGFR2 (Sc-504; WB 1:500), and PKC-ζ (Sc-17781; WB 1:500) were purchased from Santa Cruz Biotechnology. Verapamil (V4629; used at 100 μM) was purchased from Sigma. Dasatinib (D-3307; used at 500 nM) was purchased from LC Laboratories.

### Establishment of UCAFm, UCAPm, and UCAPe cells and short tandem repeat DNA fingerprinting

Human ascitic fluid was collected from a bladder cancer patient who had previously been treated with systemic chemotherapy followed by surgical extirpation of the primary tumor and regional nodal metastases (ascitic fluid specimens were collected under protocols LAB03–0320 and LAB96-178 approved by the Institutional Review Board of The University of Texas MD Anderson Cancer Center: These protocols include samples collected under an informed consent. The manuscript does not contain any information or images that could lead to identification of a study participant; all methods were performed in accordance with the institutional guidelines and regulations). Note that ascites formation in bladder cancer patients is a rare event. The ascitic fluid was centrifuged at 3500 r.p.m. for 5 minutes to obtain pellet (UCAPem) and flocculate (UCAFm) cells. 80% confluent UCAPem cells in T-75 flasks were exposed to 2 ml of trypsin-PBS-EDTA for 4 minutes (with the flask tilted at 2 minutes to prevent drying) in an incubator to dislodge mesenchymal (UCAPm) cells. Remaining adherent cells were further trypsinized with fresh trypsin for an additional 1 minute with gentle tapping of the flask against hard substratum to dislodge trypsin-resistant epithelial (UCAPe) cells. All cells were cultured in MEM with 10% fetal bovine serum, L-glutamine (MEM component), pyruvate, nonessential amino acids, vitamins, penicillin, and streptomycin.

Cells were cultured in MEM for 48 hours, and DNA was isolated using a Qiagen DNA isolation kit before it was subjected to short tandem repeat fingerprinting. The fingerprint results were unique from known cell line fingerprints and were similar among the three cell lines tested because of the origin from single patient (Supplemental Figure 1a).

### Tumorigenicity assays

For tumorigenicity assays, 200,000 cells in 100 μl of HBSS with or without matrigel (1:1) were injected subcutaneously in the flanks of male, athymic nude mice (NCr-nu/nu: NCI) (n = 5/group; was able to give statistically significant differences). Tumor formation dates were recorded; tumor growth was measured and tumor volume was calculated as described previously^42^; and overall survival was noted. See the statistical analysis section for more details. All animal experiments were done per Institutional Animal Care and Use Committee guidelines of The University of Texas MD Anderson Cancer Center, and per approved protocols.

### Microarray analysis of transcriptomes and validation

Transcriptome data were generated from total RNA isolated from UCAFm, UCAPm, and UCAPe cells (MirVana kit, Ambion) as described previously^11^. EMT phenotypes were verified using gene set variation analysis and gene set enrichment analysis. Gene set variation analysis was used to compute sample-wise gene set enrichment scores for UCAFm, UCAPm, and UCAPe cells with the epithelial or mesenchymal gene set signatures of generic cancer, generic cell lines, and bladder cancer^6^. A positive score indicated that the reference gene set matched our cell line phenotype. For gene set enrichment analysis, the ratios of UCAFm to UCAPe or UCAPm to UCAPe were used to rank all the genes in the array to compare with established epithelial or mesenchymal gene set signatures of generic cancer, generic cell lines, and bladder cancer^6^. Further, statistical significance of the gene set enrichment was tested using Fisher’s exact test. Cut-off points were set at value <−2 for epithelial and >2 for mesenchymal scores. EMT-related genes were selected from the literature (see Supplemental tables for references) to experimentally verify the top matches of microarray data and known EMT signature. Once verified, the data were validated using surface and/or total immunofluorescence, ELISA, and Western immunoblotting. Then the microarray data were used to generate stemness, calcium signaling, immunity, and top UCAPe-discriminating gene signatures for further experimental validation. For top UCAPe-discriminating gene signatures, the following criteria were used. Quantile-normalized genes were filtered using SD gene vector set at 2750, 4 duplicates were eliminated, gene expression values were log-transformed, median-centered, clustered, and average-linked using Gene Cluster 3.0 before heat maps were generated using Java TreeView version 1.1.6r2. For any genes with multiple probes, an average value was used for heat map generation except for the top 48 UCAPe-discriminating genes.

### Bright field microscopy, surface immunofluorescence, and/or total immunofluorescence microscopy and FACS

Cells were plated in 6-well or 24-well plates at a density of 50, 000 cells/ml. At 24 hours after plating, cells were treated as indicated in figures (and/or as per the reagents section) or medium was changed to allow further 24-hour incubation. For immunofluorescence microscopy, the medium was aspirated out, and cells were immediately fixed using freezing-cold methanol at −20 °C for more than 24 hours to completely disable any exocytosis or endocytosis. For FACS, cells were briefly trypsinized until they were dislodged from plates and were then pelleted down at 3500 r.p.m. for 5 minutes, and the pellets were resuspended in freezing-cold methanol and stored at −20 °C for more than 24 hours. For both microscopy and FACS, the cells were washed in PBS-BSA (1% BSA in PBS) solution, blocked in 1% BSA in PBS for surface immunofluorescence (1% BSA in PBS with 0.3% Triton-X100 for total immunofluorescence) for 30 minutes at 4 °C. Primary antibodies were incubated with cells in blocking buffer with or without Triton-X100 depending on surface or total immunofluorescence for 40 minutes at 4 °C. Cells were washed thrice and incubated further with Cy3- or Alexa-555- or Alexa-488-conjugated secondary antibodies for 1 hour at room temperature, washed thrice with PBS, and then subjected to FACS or fluorescence microscopy. For propidium iodide (PI) counterstain, the last wash in PBS was supplemented with 1:5 PBS-diluted PI-FACS solution (see DNA fragmentation analysis section).

### Quantitative ELISA

UCAFm, UCAPm, and UCAPe cells were plated at equal densities, media were changed once at 48 hours, and then the conditioned media were collected at 24 hours and clarified at 3500 r.p.m. for 5 minutes, and the supernatants without dilution were subjected to quantitative IL-8 and IL-6 ELISA per the manufacturer’s instructions.

### Inhibition of calcium signaling and detection of cell death in transformed spheres

UCAPe cells were treated with or without fresh verapamil (100 μM) plus dasatinib (500 nM) for 24 hours and then incubated for 20 minutes in 100 nM calcein-AM plus 8 μM ethidium homodimer-1 in existing MEM at room temperature [Live/Dead cytotoxicity kit (L3224); Invitrogen] before imaging.

### Western immunoblotting, secretory VEGF-A analysis, and densitometry

Western immunoblotting was performed as described previously^15^. Briefly, cells were lysed using whole cell lysis buffer [50 mM Tris-HCl, pH 7.4; 150 mM NaCl; 5 mM EDTA; 25 mM NaF; 1% Triton-X 100; 1% NP-40; 0.1 mM Na_3_VO_4_; 12.5 mM β-glycerophosphate; 1 mM PMSF, and complete protease inhibitor cocktail (Roche)] by incubation in ice for 30 to 40 minutes with intermittent vortexing every 10 minutes. The lysates were clarified at 13,000 r.p.m. for 10 minutes, and the supernatants were quantified and subjected to SDS-PAGE and Western blotting on nitrocellulose membranes. No internal lanes were cropped within displayed blot images.

A total of 200,000 cells/ml in MEM were plated for 24 hours, the conditioned medium was collected and clarified at 13,000 rpm for 10 minutes, and then 40 μl each of conditioned media or control MEM was subjected to Western blotting.

### Immunoprecipitation

RalBP1 antibody 2 μl/reaction was conjugated to protein-A sepharose CL4B beads for 30 minutes at 4 °C in a rotating platform before washing three times with whole cell lysis buffer (composition described in the Western immunoblotting section) and incubated with 200 μg/200 μl cell lysates in whole cell lysis buffer as indicated in figures) for 1hr at 4 °C. Immunoprecipitates were washed 4 times with whole cell lysis buffer before being subjected to SDS-PAGE and Western immunoblotting. The prey protein bands were normalized using RalBP1 (bait) using ImageJ software–based densitometry. Protein interaction data have been submitted to the IMEx (http://www.imexconsortium.org) consortium through IntAct^43^ and assigned the identifier IM‐24526.

### ROS measurement

Cells were plated at a density of 50,000 cells/ml and 4 ml/well of 6-well plates. Twenty-four hours later, cells were treated as indicated in the figures for 23.5 hours. At this point, 10 μM DCF-DA (2′,7′-dichlorofluorescein diacetate) was added to all wells, and cells were incubated for a further 30 minutes before analysis of ROS-activated DCF-DA fluorescence (FL-1/525 nm) by FACS (Beckman Coulter, FC500)^44, 45^. The histograms were merged using Flowjo software.

### Cell cycle and DNA fragmentation analysis

Cells were plated in triplicate at 50,000 cells/ml in complete MEM, media changed at 24 hour, and were subjected to PI-FACS at 48 hour after plating because these cells showed a lag period up to 48 hours before proliferation. PI solution (50 ng/ml PI; 1 mg/ml tri-sodium citrate; 1 μl/ml triton-X100; in PBS) was incubated with cells for 40 minutes at 4 °C for adequate staining before analysis.

### Calculation of doubling time and proliferation rate

Cells were plated in triplicate at 50,000 cells/ml in complete MEM, and the cells were trypsinized and counted at 24 hours, 48 hours, and 72 hours. The doubling time was calculated using a web-based doubling time calculator (http://www.doubling-time.com/compute.php) (Supplemental Figure 1b). The cell densities at these time points were plotted to compare the proliferation rates (Supplemental Figure 1c).

### Statistical analyses

Statistical analyses were performed using Microsoft Excel 2010. Statistical significance was determined based on Student’s *t*-test with two-tailed distribution and two-sample unequal variance. Error bars represent standard errors of the mean. For survival and tumorigenesis experiments, GraphPad Prism 5 software was used with log-rank test for evaluating significance. *P* values below 0.05 were considered significant. Please see the microarray data section for statistical analysis of gene set enrichment data.

## Electronic supplementary material

Supplemental Figures and Tables
