# Supplementary material for: Surface PD-L1, E-cadherin, CD24, and VEGFR2 as markers of epithelial cancer stem cells associated with rapid tumorigenesis
Source: Sci Rep. 2017 Aug 29;7:9602. doi: 10.1038/s41598-017-08796-z (PMC5575243; doi:10.1038/s41598-017-08796-z)
Supplement: Supplementary file 1 — Supplemental Figures and Tables [file 41598_2017_8796_MOESM1_ESM.pdf]

**Surface E-cadherin, CD24, PD-L1, and VEGFR2 as markers of migrating epithelial cancer stem cells associated with rapid tumorigenesis**

Goodwin G. Jinesh, Ganiraju C. Manyam, Chinedu O. Mmeje, Keith A. Baggerly, and Ashish M. Kamat

## Supplemental information

**Supplemental figure - 1**

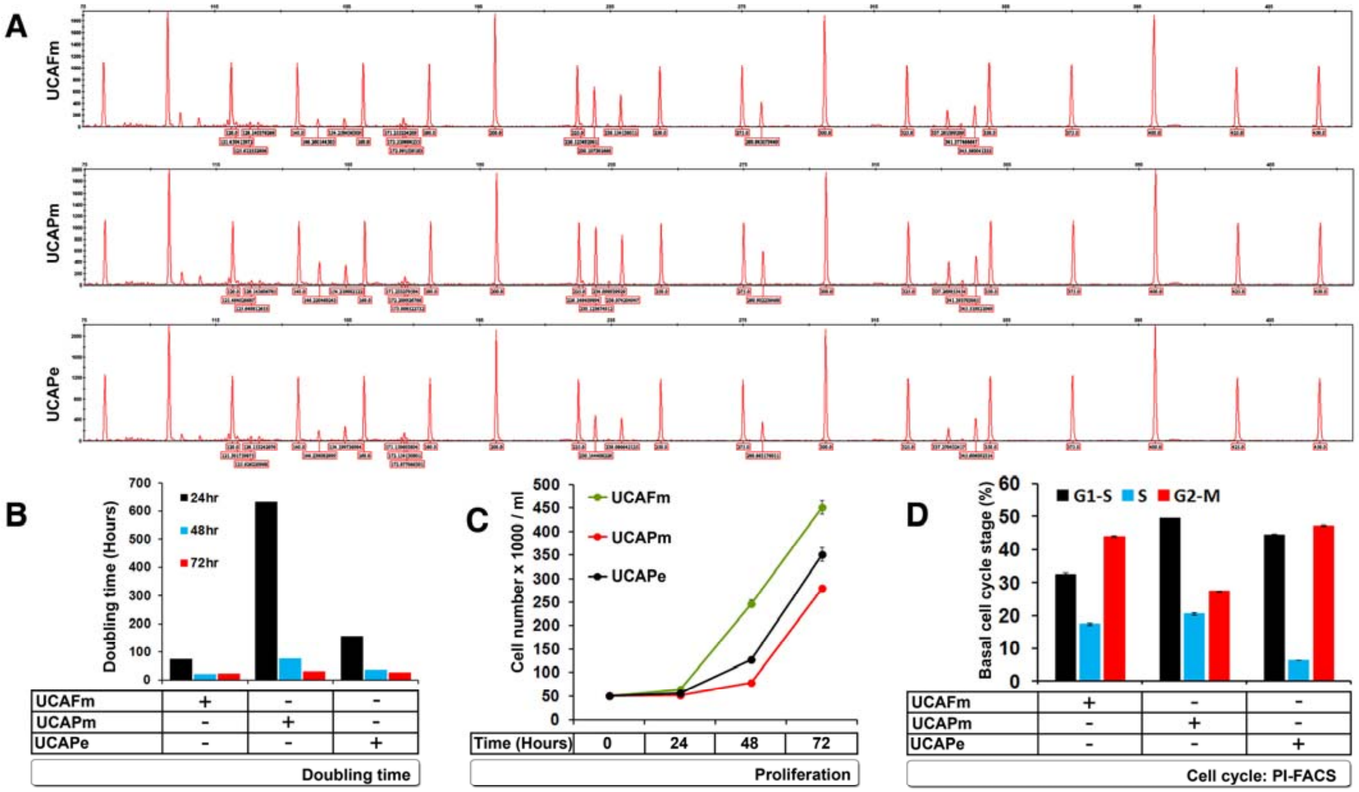


**Supplemental Figure 1. Characteristic features of newly established UCAFm, UCAPm, and UCAPe cell lines**

**A.** Short tandem repeat (STR) DNA fingerprinting of UCAFm, UCAPm, and UCAPe cells showing similar STR profiles because of the origin from the same patient. **B.** Doubling time calculation of UCAFm, UCAPm, and UCAPe cells showing that UCAPm cells had no proliferation in the first 24 hours after trypsinization but all three cell lines had fast and similar doubling times from 48 through 72 hours after trypsinization. **C.** Cell proliferation analysis of UCAFm, UCAPm, and UCAPe cells showing a lag period up to 48 hours before cells started proliferating at a similar pace. **D.** Cell cycle analysis by PI-FACS at 48 hours after trypsinization, when the cells showed the greatest difference in proliferation (see panel **C**).

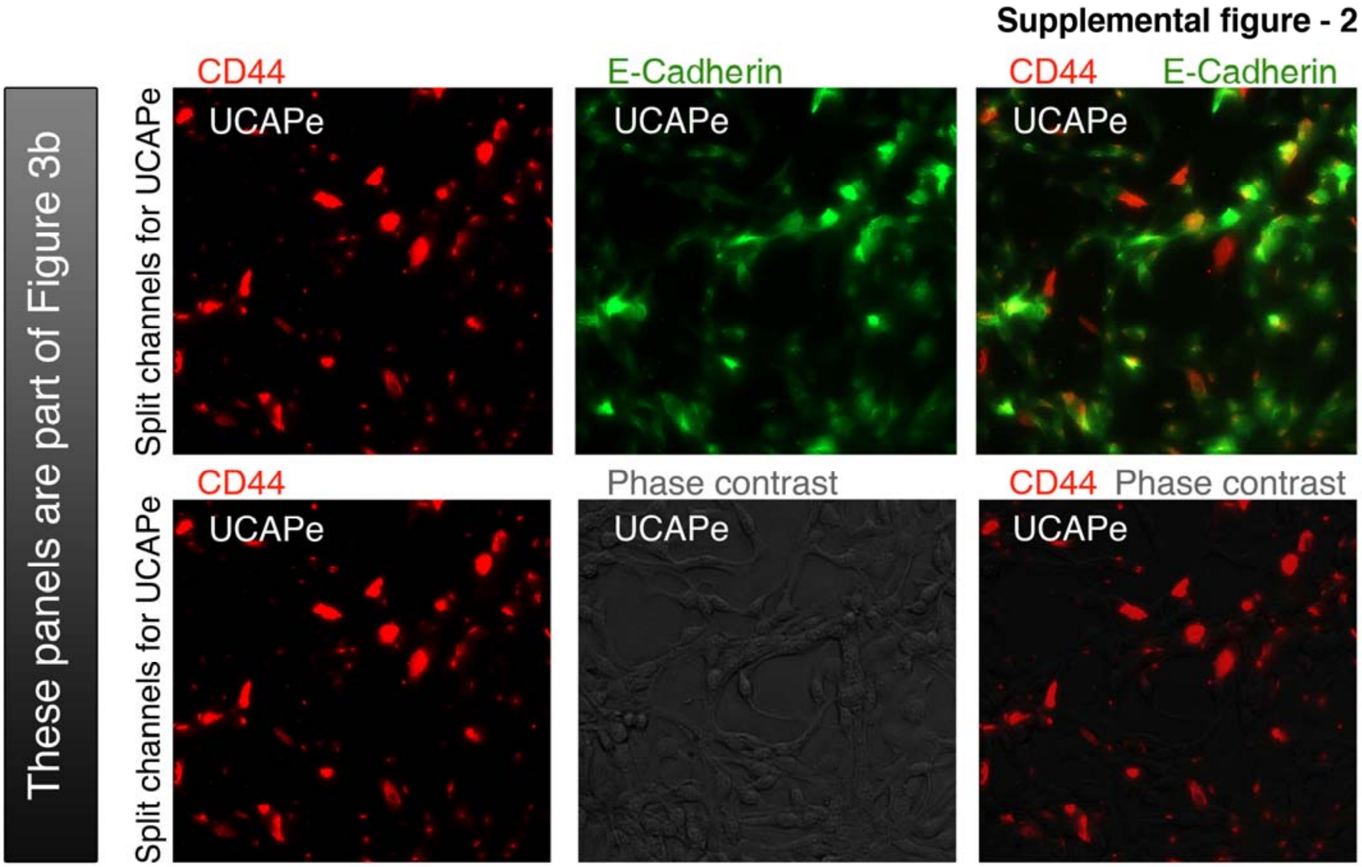


**Supplemental Figure 2. Majority of UCAPe cells express CD44**

These single channel panels (part of the main figure 3b; surface immunofluorescence of surface CD44 versus surface E-cadherin) are shown to demonstrate that CD44 is expressed in most UCAPe cells (top left panel) but appears less in intensity due to E-cadherin positivity (top right panel). Hence the surface CD44 data on main figure 3b (surface immunofluorescence microscopy) and 5c (surface immunofluorescence FACS to count number of positive cells) should be interpreted with caution.

**Supplemental table-1: Expression of known EMT regulator genes among UCAFm, UCAPm and UCAPe transcriptomes**

| Target_ID | UCAFm | UCAPm | UCAPe | Functional description (relevant to this study/known functions) | Reference(s) |
|---|---|---|---|---|---|
| CD24 | 137.9 | 434.7 | 7193.8 | Cancer Stem Cell Marker; Induces Calcium flux; Cell adhesion; Promotes antigen-dependent proliferation of B-cells, and prevents their terminal differentiation into antibody-forming cells. | Eur J Immunol. 1993 Nov;23(11):2782-91; PLoS One, v9: page e109495 |
| CDH1 | 108.3 | 459.3 | 2274.6 | E-Cadherin is an epithelial marker | Genes & Dev: v22: p894 |
| CDH15 | 1320.3 | 852.7 | 142.2 | calcium-dependent cell adhesion protein | Experimental cell research, v232: p331-8 |
| CDH2 | 108.5 | 192.8 | 1839.8 | Overexpression of N-cadherin is involved in EMT (Ref:CCR) but can also expressed in epithelial cells with invasion (Ref: IJDB) | Clin Can Res: v10: p4125; Int J Dev Biol: v48: p463 |
| CLDN7 | 149.0 | 320.4 | 1853.3 | EpCAM-claudin-7 complex strongly promotes tumorigenicity, accelerates tumor growth, and supports ascites production | Mol Can Res: v7: p285 |
| CSNK1A1 | 295.9 | 275.3 | 238.2 | Casein Kinase-1 negatively regulates E-Cadherin based cell-cell contacts by phosphorylation of E-cadherin at Ser-846 | Mol Cell Biol: v27: p3804 |
| CTNNBIP1 | 127.3 | 134.2 | 204.9 | TGF-beta signaling targets expression of CTNNBIP1 through miRNAs | PLoSOne v8: e58622 |
| DAB2 | 810.1 | 535.4 | 146.4 | Disabled homolog-2 is required for recycling TGF-beta receptor-II (TGFBR2 mediates EMT) | Mol Biol Cell: v21: p4009 |
| EPCAM | 1117.5 | 1691.0 | 5616.2 | TACSTD1, a human tumor-associated antigen promotes Th2 development and tumor immune evasion; an epithelial marker; with CD44 identify tumor initiating cells | Curr Mol Med: v8: p784; Blood 113:3494-502 |
| IFNGR2 | 3839.8 | 3776.6 | 2782.4 | Mutant IFNGR2 leads to susceptibility to BCG and non-TB mycobacteria and high IgE levels in serum | http://www.uniprot.org/uniprot/P38484 ; BBRC v263: p425-9 |
| IL6 | 3233.5 | 3621.8 | 217.6 | Interleukin-6 induces EMT; autoimmunity, chronic inflammation and inflammation-associated cancer; differentiation of B-cells into Ig-secreting cells; Acts on B-cells, T-cells | Oncogene vol 28: page 2940; Cytokine Growth Factor Rev. v22: p83 |
| IL8 | 7322.9 | 8903.3 | 1399.1 | Linked to EMT; IL-8 attracts neutrophils, basophils, and T-cells, but not monocytes. It is also involved in neutrophil activation | Exp Cell Res: v299: p315; Future Oncol v8: p713 |
| ITGAV | 1361.0 | 938.2 | 834.6 | Required for TGF-beta induced EMT | Cell cycle; v9: p1647 |
| KLK6 | 108.4 | 413.6 | 2668.1 | Calcium flux; cellular transformation; cell migration, invasion, inhibits EMT | J Invest Dermatol. v131:p2281; and Cancer Research v69: p3779 |
| LTBP3 | 166.5 | 178.7 | 245.2 | Latent TGF-beta binding protein-3 blocks TGF-beta activation at ECM (thereby it can block TGF-beta mediated EMT) | J Biol Chem: v270: 10147 |
| NUMBL | 212.1 | 155.8 | 141.2 | (NUMB ligand can activate) NUMB mediates endocytosis of E-Cadherin | Mol Biol Cell: v22: p3103 |
| PRKCZ | 103.6 | 108.6 | 156.2 | PKC-zeta is a tight junction associated kinase phosphorylates ZO-1, occludin and claudin-1 to mediate cell adhesion; mediates glucose induced VEGF expression | J Cell Biol: v158: p967; American journal of physiology. Endocrinology and metabolism, v293: page E1280 |
| PRSS8 | 117.7 | 216.9 | 912.4 | Loss of PRSS8 leads to EMT in bladder cancer | BMC Cancer, v9, p377 |
| RAP1GAP | 101.9 | 108.8 | 182.4 | RAP1GAP activates Rap1 to mediate E-cadherin based cell-cell contacts | Mol Cell Biol: v22: p6690 |
| TGFBR2 | 435.3 | 417.3 | 260.8 | mediates TGFb induced EMT; Immune evasion of cancer; alters DC regulated Treg cell phenotypes | Cancer Cell : 8:369-80; J Immunol. V189: p3878; British journal of cancer, v94: p661 |
| TGFBR3 | 362.1 | 405.0 | 135.5 | Upregulated during EMT | Molecular cancer; v12: p90 |
| TWIST1 | 794.1 | 551.4 | 224.8 | TWIST is a transcriptional repressor of E-Cadherin | Biochem Biophys Res Commun: v367: p235 |
| VEGFC | 1855.8 | 1567.8 | 567.5 | VEGF-C downregulation restores epithelial phenotype | Oncogene, v31, p1389 |
| ZEB2 | 388.4 | 279.6 | 110.5 | ZEB2 repress E-Cadherin expression | Genes & Dev: v22: p894 |

**Supplemental table 2: Expression of known stemness-related genes among UCAFm, UCAPm and UCAPe transcriptomes**

| Target_ID | UCAFm | UCAPm | UCAPe | Functional description of encoded protein (relevant to this study/known functions) | Reference(s) |
|---|---|---|---|---|---|
| **AKR1B1** | 6170.3 | 5352.7 | 1083.5 | Works against ALDH by reducing substrates; Lower expression is associated with malignancy | J Clin Endocrinol Metab volume 89: page 3010 |
| **ALDH1A3** | 918.8 | 2599.0 | 6157.1 | Cancer stem cell marker | Stem Cells volume 29: page 32 |
| **CD24** | 137.9 | 434.7 | 7193.8 | Cancer stem cell Marker; Induces Calcium flux; Cell adhesion; Promotes antigen-dependent proliferation of B-cells and prevents their terminal differentiation into antibody-forming cells. | Eur J Immunol. 1993 Nov;volume 23(11):page 2782-91; PLoS One, volume 9: page e109495 |
| **CD44** | 3019.1 | 3937.1 | 1673.2 | Cancer stem cell marker; Also marks normal stem cells | Exp Biol Med, volume 238:page 324-38 |
| **KITLG** | 131.3 | 200.6 | 377.7 | Ligand for c-Kit | Oncotarget. Volume 5: page 10486–10502 |
| **KRT19** | 1364.4 | 1645.4 | 9313.8 | Cancer stem cell marker in hepatocellular carcinoma and colon cancer | Cell Stem Cell volume 16, page 627–638; Clin Cancer Res.volume 21: page 3081-91 |
| **SOX4** | 268.9 | 455.5 | 2126.5 | Positive regulator of apoptosis | Experimental & molecular medicine volume 34: page 243 |

**Supplemental table 3: Expression of calcium signaling related genes among UCAFm, UCAPm and UCAPe transcriptomes**

| Target_ID | UCAFm | UCAPm | UCAPe | Functional description of encoded protein (relevant to this study/known functions) | Reference(s) |
|---|---|---|---|---|---|
| CAMK2N1 | 183.1 | 478.2 | 5267.4 | Inhibits complement factor B-induced membrane injury by inhibiting calcium/calmodulin-dependent protein kinase II | J Clin. Investigation, volume 119: page 986; http://www.uniprot.org/uniprot/Q7Z7J9 |
| CD24 | 137.9 | 434.7 | 7193.8 | Cancer stem cell marker; Induces calcium flux; Cell adhesion; Promotes antigen-dependent proliferation of B-cells, and prevents their terminal differentiation into antibody-forming cells. | Eur J Immunol. volume 23(11):page 2782-91; PLoS One, volume 9: page e109495 |
| EPCAM | 1117.5 | 1691.0 | 5616.2 | TACSTD1, a human tumor-associated antigen promotes Th2 development and tumor immune evasion; An epithelial cell marker; With CD44 identifies tumor-initiating cells | Curr Mol Med: volume 8: page 784; Blood volume 113:page 3494-502 |
| PLA2G10 | 121.0 | 286.4 | 3975.0 | Has a powerful potency for releasing arachidonic acid from cell membrane phospholipids | http://www.uniprot.org/uniprot/O15496 |
| S100A10 | 7273.6 | 6046.3 | 15430.1 | Plasminogen receptor; Attracts tumor associated macrophages (TAMs) to tumor microenvironment | Cancer Res, volume 71;page 6676 |
| S100P | 987.9 | 2557.2 | 12932.2 | Calcium binding protein associated with polyploid tumors | International journal of molecular medicine volume 35: page 675-83 |
| TACSTD1 | 956.0 | 1408.6 | 5427.3 | TACSTD1, a human tumor-associated antigen promotes Th2 development and tumor immune evasion; An epithelial cell marker; With CD44 identifies tumor initiating cells | Curr Mol Med: volume 8: page 784; Blood volume 113:page 3494-502 |
| TACSTD2 | 2331.2 | 3085.6 | 11912.9 | TROP-2, a tumor-associated calcium signal transducer | Int J Cancer volume 76: page 671-6 |

**Supplemental table 4: Expression of tumor immunology-related genes among UCAFm, UCAPm and UCAPe transcriptomes**

| TargetID_Probe ID | UCAFm | UCAPm | UCAPe | Functional description of gene products (relevant to this study/known functions) | Reference(s) |
|---|---|---|---|---|---|
| **BGN** | 8395.7 | 9031.0 | 1566.6 | Induced in response to inflammation; TGF-beta target gene (might act during resolution phase of inflammation) | The Journal of biological chemistry; volume 278, page 11041 and The Journal of biological chemistry; volume 277, page 36118 |
| **CAMK2N1** | 183.1 | 478.2 | 5267.4 | Inhibits complement factor B-induced membrane injury by inhibiting calcium/calmodulin-dependent protein kinase II | J Clin. Investigation, volume 119: page 986; http://www.uniprot.org/uniprot/Q7Z7J9 |
| **CCL20** | 1589.5 | 1622.7 | 101.6 | Chemotactic factor that attracts lymphocytes and, slightly, neutrophils, but not monocytes | http://www.uniprot.org/uniprot/P78556 |
| **CD24** | 137.9 | 434.7 | 7193.8 | Cancer stem cell marker; Induces calcium flux; Cell adhesion; Promotes antigen-dependent proliferation of B-cells, and prevents their terminal differentiation into antibody-forming cells. | Eur J Immunol. volume 23(11):page 2782-91; PLoS One, volume 9: page e109495 |
| **CD274** | 106.7 | 130.4 | 212.2 | PD-L1; involved in counter attack of immune cells; essential for T-cell proliferation and production of IL-10 and IFNgamma | http://www.uniprot.org/uniprot/Q9NZQ7 |
| **CEBPD** | 4561.2 | 4019.1 | 988.6 | Regulation of the several genes associated with activation and/or differentiation of macrophages | http://www.uniprot.org/uniprot/P49716 |
| **CFB** | 5331.6 | 6302.1 | 584.3 | Induced cell membrane injury | http://www.uniprot.org/uniprot/P00751 |
| **CFD** | 187.7 | 129.8 | 102.3 | Cleaves and activates Complement Factor-B | http://www.uniprot.org/uniprot/P00746 |
| **CFH** | 1015.7 | 1058.2 | 200.0 | Functions as a cofactor in the inactivation of C3b by factor I and also increases the rate of dissociation of the C3bBb complex (C3 convertase) and the (C3b)NBB complex (C5 convertase) in the alternative complement pathway | http://www.uniprot.org/uniprot/P08603 |
| **CSF2** | 760.3 | 664.4 | 108.1 | GM-CSF; Stimulates the growth and differentiation of hematopoietic precursor cells from various lineages, including granulocytes, macrophages, eosinophils and erythrocytes | http://www.uniprot.org/uniprot/P04141 |
| **CXCL1** | 3916.3 | 5911.5 | 511.2 | Has chemotactic activity for neutrophils | J Interferon Cytokine Res, volume 29:page 657-66 |
| **CXCL6** | 2771.4 | 6326.3 | 308.7 | Chemotactic for neutrophil granulocytes. Signals through binding and activation of its receptors (CXCR1 and CXCR2). | Exp Cell Res, volume 303:page 331-42 |
| **EPCAM** | 1117.5 | 1691.0 | 5616.2 | TACSTD1, a human tumor-associated antigen promotes Th2 development and tumor immune evasion; An epithelial cell marker; With CD44 identifies tumor initiating cells | Curr Mol Med: volume 8: page 784; Blood volume 113:page 3494-502 |
| **EZR** | 3798.2 | 4411.4 | 9985.2 | Ezrin tunes the magnitude of humoral immunity | J Immunol, volume 191:page 4048-58 |
| **HLA-A** | 9982.5 | 10170.5 | 6807.9 | Antigen presentation to immune system | http://www.uniprot.org/uniprot/P01892 |
| **HLA-B** | 10353.8 | 9731.4 | 3203.6 | Antigen presentation to immune system | http://www.uniprot.org/uniprot/P18464 |
| **HLA-F** | 2828.2 | 2740.3 | 1086.9 | Antigen presentation to immune system | http://www.uniprot.org/uniprot/P30511 |

| | | | | | |
|---|---|---|---|---|---|
| **HLA-H** | 7442.2 | 7567.8 | 3000.8 | Antigen presentation to immune system | http://www.uniprot.org/uniprot/P01893 |
| **IL1B** | 1112.8 | 1847.2 | 122.1 | Induces IL-2 release, B-cell maturation and proliferation, and fibroblast growth factor activity. IL-1 proteins are involved in the inflammatory response | http://www.uniprot.org/uniprot/P01584 |
| **IL6** | 3233.5 | 3621.8 | 217.6 | Interleukin-6 induces EMT, autoimmunity, chronic inflammation and inflammation-associated cancer; Differentiation of B-cells into Ig-secreting cells; Acts on B-cells, T-cells | Oncogene volume 28: page 2940; Cytokine Growth Factor Rev. volume 22: page 83 |
| **IL8** | 7322.9 | 8903.3 | 1399.1 | Linked to EMT; IL-8 attracts neutrophils, basophils, and T-cells, but not monocytes. It is also involved in neutrophil activation | Exp Cell Res: volume 299: page 315; Future Oncol volume 8: page 713 |
| **ITGAE** | 624.2 | 736.8 | 1093.0 | CD103; E-cadherin's ligand on DC, T and NK cells mediates E-cadherins calcium dependent interaction of immune cells | Blood volume 119: page 1623; Curr Opin Cell Biol. volume 12: page 563 |
| **KRT17** | 172.8 | 250.7 | 927.3 | Promotes Th1/Th17-dominated immune environment contributing to the development of basaloid skin tumors | http://www.uniprot.org/uniprot/Q04695 |
| **LCN2** | 503.6 | 7122.0 | 7793.6 | Innate immunity against bacterial infections by sequestering iron | http://www.uniprot.org/uniprot/P80188 |
| **LCP1** | 172.9 | 126.7 | 1447.2 | The Actin-Bundling Protein L-Plastin is a critical regulator of immune cell function by activation of T-cells | Int J Cell Biol volume 2012: Article ID: 935173 |
| **LGALS1** | 10972.6 | 8529.8 | 6565.9 | Galectin-1; Strong inducer of T-cell apoptosis [Explains why it did not help HAFm cells in athymic nude mice which lack functional T-cells] | Cancer Res,  volume 71;  page 4423 |
| **LOX** | 1243.3 | 742.0 | 122.6 | It acts as a receptor for the HSP70 protein involved in antigen cross-presentation to naive T-cells in dendritic cells, thereby participating in cell-mediated antigen cross-presentation, related to transformation | Mol Cell Biol. volume 31:page 2683-95 |
| **PLA2G10** | 121.0 | 286.4 | 3975.0 | Has a powerful potency for releasing arachidonic acid from cell membrane phospholipids | http://www.uniprot.org/uniprot/O15496 |
| **PLAT** | 462.1 | 2093.1 | 6615.7 | Plasminogen activator [See S100A10 description] | http://www.uniprot.org/uniprot/P00750 |
| **S100A10** | 7273.6 | 6046.3 | 15430.1 | Plasminogen receptor - attracts tumor associated macrophages (TAMs) to tumor microenvironment | Cancer Res, volume 71;page 6676 |
| **SERPINB2** | 639.1 | 313.5 | 112.8 | Inhibits urokinase-type plasminogen activator. The monocyte derived PAI-2 is distinct from the endothelial cell-derived PAI-1 | http://www.uniprot.org/uniprot/P05120 |
| **SERPINE1** | 3142.4 | 2885.1 | 342.5 | PAI; TGFb target; This inhibitor acts as 'bait' for tissue plasminogen activator, urokinase, protein C and matriptase-3/TMPRSS7. | http://www.uniprot.org/uniprot/P05121 |
| **SPP1** | 102.1 | 203.5 | 1371.3 | Osteopontin; Acts as a cytokine involved in enhancing production of interferon-gamma and interleukin-12 and reducing production of interleukin-10 and is essential in the pathway that leads to type I immunity | http://www.uniprot.org/uniprot/P10451 |
| **SRGN** | 8304.7 | 6927.8 | 3389.7 | Regulates protease storage in secretory granules such as Granzyme-B, elastase etc. | Immunity, volume 16:page 417-28 |
| **TACSTD1** | 956.0 | 1408.6 | 5427.3 | TACSTD1, a human tumor-associated antigen promotes Th2 development and tumor immune evasion; an epithelial marker; with CD44 identify tumor initiating cells | Curr Mol Med: volume 8: page 784; Blood volume 113:page 3494-502 |

| | | | | | |
|---|---|---|---|---|---|
| **TGFBR2** | 435.3 | 417.3 | 260.8 | Mediates TGFb induced EMT; Immune evasion of cancer; Alters DC regulated Treg cell phenotypes | Cancer Cell : volume 8:page 369-80; J Immunol. volume 189: page 3878; British journal of cancer, volume 94: page 661 |
| **TXNIP** | 383.4 | 1746.6 | 6986.0 | Required for the maturation of natural killer cells | http://www.uniprot.org/uniprot/Q9H3M7 |

## Supplemental table 5: Rapid tumorigenesis signature: top 48 genes that discriminate UCAPe cells from UCAFm, and UCAPm cells

| TargetID | UCAFm | UCAPm | UCAPe | Functional description (relevant to this study/known functions) | Reference(s) |
|---|---|---|---|---|---|
| ACTB | 8749.5 | 11698.4 | 15652.3 | Multiple functions including transformation, cytoskeleton, and cell adhesion. | Clin Chim Acta volume 417: page 39 |
| ACTG1 | 5044.7 | 8055.3 | 12441.0 | ACTG1 inhibits cell migration | Mol Med Reports volume 9: page 387 |
| ALDH1A3 | 1119.5 | 3280.8 | 7934.4 | Cancer stem cell marker | Stem Cells volume 29: page 32 |
| ANXA2 | 12565.2 | 10681.0 | 17638.1 | Cell adhesion; Migration of cancer cells | J Cellular Biochem. volume 105: page 370 |
| ARHGDIB | 134.1 | 1361.5 | 5798.9 | Rho GDP-dissociation Inhibitor 2 [Negatively regulates Rho signaling] | AIDS research and human retroviruses; volume 28, page 913 |
| ARMET | 8838.5 | 2977.8 | 2809.6 | Unfolded protein response (Autophagy) | Cell Struct Funct. volume 32: page 41 |
| BCYRN1 | 9868.4 | 9707.0 | 4924.2 | BCYRN1 (brain cytoplasmic RNA 1) is an RNA gene, and is affiliated with the lncRNA class | http://www.genecards.org/cgi-bin/carddisp.pl?gene=BCYRN1 |
| BGN | 8395.7 | 9031.0 | 1566.6 | Induced in response to inflammation; TGF-beta target gene [might act during resolution phase of inflammation] | The Journal of biological chemistry; volume 278, page 11041 and The Journal of biological chemistry; volume 277, page 36118 |
| BOLA2 | 5479.0 | 6944.8 | 10927.7 | Poorly characterized protein | http://www.uniprot.org/uniprot/Q9H3K6 |
| CAMK2N1 | 183.1 | 478.2 | 5267.4 | Inhibits Complement factor B-induced membrane injury by inhibiting calcium/calmodulin-dependent protein kinase II | J Clin. Investigation, volume 119: page 986; http://www.uniprot.org/uniprot/Q7Z7J9 |
| CD24 | 177.6 | 694.6 | 12565.2 | Cancer stem cell marker; Induces calcium flux; Cell adhesion; Promotes antigen-dependent proliferation of B-cells, and prevents their terminal differentiation into antibody-forming cells. | Eur J Immunol. volume 23(11):page 2782-91; PLoS One, volume 9: page e109495 |
| CD44 | 7598.0 | 10201.9 | 4426.5 | Cancer stem cell marker and also marks normal stem cells | Exp Biol Med, volume 238:page 324-38 |
| CFB | 5331.6 | 6302.1 | 584.3 | Complement factor B, induces cell membrane injury | http://www.uniprot.org/uniprot/P00751 |
| CLDN11 | 6637.1 | 7145.6 | 281.2 | CLDN11 hypermethylation marks malignancy | The Journal of investigative dermatology, volume 134: page 2957-66 |
| CXCL6 | 3845.2 | 8362.5 | 387.4 | Chemotactic for neutrophil granulocytes. Signals through binding and activation of its receptors (CXCR1 and CXCR2). | Exp Cell Res, volume 303:page 331-42 |
| EZR | 3798.2 | 4411.4 | 9985.2 | Ezrin tunes the magnitude of humoral immunity | J Immunol, volume 191:page 4048-58 |
| FHL2 | 7749.1 | 5786.8 | 11295.9 | FHL2 interferes with the transcriptional activity of FOXO1 and the expression of known FOXO target genes and inhibits FOXO1-induced apoptosis | EMBO J. volume 24:page 1021-32 |
| FTL | 19365.8 | 17181.9 | 13034.1 | Stores iron in a soluble, non-toxic, readily available form. | http://www.uniprot.org/uniprot/P02792 |
| H3F3A | 8157.0 | 11255.6 | 14223.6 | Regulation of chromating activity states | Development, volume 140, page 3624-3634 |
| HLA-A | 15400.2 | 17793.6 | 12162.8 | Antigen presentation to immune system | http://www.uniprot.org/uniprot/P01892 |
| HLA-B | 10353.8 | 9731.4 | 3203.6 | Antigen presentation to immune system | http://www.uniprot.org/uniprot/P18464 |
| HLA-H | 11374.7 | 12162.8 | 4637.1 | Antigen presentation to immune system | http://www.uniprot.org/uniprot/P01893 |
| HSP90B1 | 11007.9 | 5616.2 | 4721.0 | Molecular chaperone process and transport secreted proteins-Functions in endoplasmic reticulum associated degradation | Nat Cell Biol. volume 10:page 272-82 |
| IGFBP3 | 5360.6 | 7560.6 | 10895.5 | Suppresses oxidative stress in hypoxic tumor microenvironment | Am J Cancer Res, volume 4:page 29-41 |
| IL8 | 7322.9 | 8903.3 | 1399.1 | Linked to EMT; IL-8 attracts neutrophils, basophils, and T-cells, but not monocytes. It is also involved in neutrophil activation | Exp Cell Res: volume 299: page 315; Future Oncol volume 8: page 713 |
| KLK6 | 114.2 | 713.8 | 5216.3 | Inhibits EMT, involved in cellular transformation, calcium flux, cell migration and invasion | J Invest Dermatol volume 131: page 2281 and Can Res volume 69: page 3779 |
| KRT19 | 1364.4 | 1645.4 | 9313.8 | It's a cancer stem cell marker in HCC and colon cancer | Cell Stem Cell volume 16, page 627–638; Clin Cancer Res. volume 21: page 3081-91 |

| | | | | | |
|---|---|---|---|---|---|
| **LCN2** | 503.6 | 7122.0 | 7793.6 | Innate immunity against bacterial infections by sequestering iron | http://www.uniprot.org/uniprot/P80188 |
| **LOC100008589** | 7123.8 | 1299.9 | 446.0 | 28S ribosomal RNA | http://refgene.com/gene/100008589 |
| **LOC100132394** | 7208.3 | 1931.2 | 490.1 | Hypothetical protein | http://www.ncbi.nlm.nih.gov/gene/100132394 |
| **LOC284393** | 17057.7 | 14238.1 | 10447.0 | Ribosomal protein L10 pseudogene 16 | http://refgene.com/gene/284393 |
| **LOC392437** | 8782.0 | 5220.1 | 3254.7 | Ferritin, light polypeptide pseudogene 2 | http://www.ncbi.nlm.nih.gov/gene?cmd=Retrieve&dopt=full_report&list_uids=392437 |
| **LOC440926** | 7237.5 | 9106.1 | 13699.1 | H3 histone, family 3A pseudogene | http://refgene.com/gene/440926 |
| **LOC644039** | 16424.3 | 11561.4 | 6035.9 | Laminin receptor homolog | http://www.ncbi.nlm.nih.gov/sviewer/viewer.fcgi?db=nuccore&val=89029934&sat=4&satkey=12861395 |
| **LOC646723** | 1560.3 | 2606.2 | 10681.0 | Type I cytoskeletal 18 (Cytokeratin-18) (CK-18) (Keratin-18) pseudogene | http://www.gene-profiles.org/gene/loc646723-homo-sapiens-646723 |
| **PLAT** | 462.1 | 2093.1 | 6615.7 | Plasminogen activator [See S100A10 description] | http://www.uniprot.org/uniprot/P00750 |
| **PRNP** | 9525.8 | 8740.1 | 3164.1 | PRNP prion protein is a target of ER stress | Breast cancer research, volume 15: page R22 |
| **RARRES1** | 5181.0 | 5588.9 | 10210.6 | Increased in response to TGF-beta1 induced liver fibrosis | Journal of molecular medicine; volume 90: page 1439-47 |
| **S100A10** | 7273.6 | 6046.3 | 15430.1 | Plasminogen receptor - attracts tumor associated macrophages (TAMs) to tumor microenvironment | Cancer Res, volume 71;page 6676 |
| **S100P** | 987.9 | 2557.2 | 12932.2 | Calcium binding protein associated with polyploid tumors | International journal of molecular medicine volume 35: page 675-83 |
| **SAT1** | 6619.4 | 13006.5 | 6373.6 | Contributes to radioresistance and present poor prognosis in GBM; offers chemoresistance in colorectal cancer cells | SciBX **7**(41); doi:10.1038/scibx.2014.1200 http://www.nature.com/scibx/journal/v7/n41/pdf/scibx.2014.1200.pdf ; Mol Cancer Ther. volume 6:page 128-37 |
| **SPINT2** | 2516.2 | 2952.2 | 8608.0 | Regulates cancer cell migration | J Invest Dermatol. volume 135:page 2283-91 |
| **SPOCK2** | 115.8 | 1062.7 | 8658.9 | Testican2; Calcium binding protein; Hypermethylated during transformation | J Ovarian Res. 2014; volume 7: page 73; J Neurochem volume 73:page 12-20 |
| **SQSTM1** | 15561.9 | 9812.8 | 5576.4 | Marks organelles for autophagy | PLoSOne volume 8: page e86017 |
| **TACSTD1** | 1679.1 | 2492.5 | 9645.7 | EPCAM; TACSTD1, a human tumor-associated antigen promotes Th2 development and tumor immune evasion; an epithelial marker; with CD44 identify tumor initiating cells | Curr Mol Med: volume 8: page 784; Blood volume 113:page 3494-502 |
| **TACSTD2** | 2331.2 | 3085.6 | 11912.9 | TROP-2, a tumor associated calcium signal transducer | Int J Cancer volume 76: page 671-6 |
| **TDP1** | 7175.7 | 11783.7 | 6592.5 | Contributes to topoisomerase inhibitor camptothecin resistance | Lung Cancer volume 55: page 303-311 |
| **TXNIP** | 383.4 | 1746.6 | 6986.0 | Required for the maturation of natural killer cells | http://www.uniprot.org/uniprot/Q9H3M7 |

Single probes for S. table-5
